# Eating Disorder Treatments Are Less Effective for Autistic  Populations: Proposing Steps Toward Improving Outcomes

**DOI:** 10.1002/eat.24335

**Published:** 2024-11-25

**Authors:** Melissa H. Black, Sven Bölte

**Affiliations:** ^1^ Center of Neurodevelopmental Disorders (KIND), Department of Women's and Children's Health Centre for Psychiatry Research, Karolinska Institutet & Region Stockholm Stockholm Sweden; ^2^ Child and Adolescent Psychiatry Stockholm Health Care Services Stockholm Sweden; ^3^ Curtin Autism Research Group Curtin School of Allied Health, Curtin University Perth Australia

**Keywords:** autism, autism spectrum conditions, co‐creation, co‐occurring conditions, intervention, participatory approaches

## Abstract

The recent mixed‐methods review by Nimbley et al. (2025) raises important and concerning, yet not unexpected, insights into the usefulness of eating disorder (ED) treatment for autistic populations. In their review, Nimbley et al. find that ED treatments may be less effective for autistic groups, proposing a need for a greater understanding of ED in autistic populations, and more autism‐informed measures and interventions for EDs. We take the opportunity in this commentary to further expand on the next steps that must be taken to inform future ED interventions for autistic populations. We reflect on similar observations of the impact of co‐occurrence on intervention efficacy in autism, draw on contemporary movements in relation to interventions in the context of autism, and align with the conclusions of Nimbley et al., who propose that future ED interventions may need to be tailored to autistic populations. We present participatory and co‐creation research approaches as a means to achieve this.

1

We find the mixed‐methods review by Nimbley et al. (2025) highly insightful and of particular importance given the detrimental impacts that untreated eating disorders (EDs) present and their apparent prevalence within autistic populations (We acknowledge that language preferences may vary. In this commentary “autistic” is used to align with language preferences of the autism community.) We are particularly interested in exploring the implications and the next steps that must be taken to address the issues raised in this review, particularly concerning the need for tailored autism‐specific ED interventions. We would like to take the opportunity to further dwell on future avenues and directions in this space.

Nimbley et al. found that autistic populations tend to spend longer in ED treatment, have more complex psychosocial difficulties that persist despite treatment, have poorer experiences of ED treatments, and report that treatments may not align with their specific needs. In sum, they conclude that current interventions for treating ED may be less effective for autistic individuals. Unfortunately, the finding that the co‐occurrence of other neurodevelopmental or psychiatric conditions may complicate both presenting difficulties and the effectiveness of treatment is not isolated to the context of EDs. In general, a greater degree of diagnostic complexity is associated with more extensive difficulties and treatment or intervention considerations. Just one example is the co‐occurrence of autism with ADHD. ADHD and autism frequently co‐occur, interfering with treatment and intervention efficacy and approaches. Here, a recent systematic review and meta‐analysis found that some medications used for treating ADHD symptoms may be less effective and result in more adverse effects among autistic individuals, highlighting the need for further investigation of treatment efficacy and safety for this group of individuals (Martins et al. 2024). Similar impacts of co‐occurring conditions with autism are also observed for other interventions, such as social skills training.

Though the co‐occurrence of multiple conditions is common, and the influence of diagnostic complexity is rather consistently observed in other contexts, these aspects are rarely addressed in clinical practice. Interventions are often focused on “pure” variants as opposed to the more complex profiles that are frequently experienced. At the same time, clinicians working within specific clinical populations are often ill‐equipped to meet the needs of individuals with more complex presentations. Autism alone is a condition in which clinicians may feel particularly unequipped, with a recent study finding that psychotherapists working with adult populations were largely unprepared to work with autistic adults, holding more misconceptions and reporting lower perceived knowledge, training, and competency in working with this population, even compared with other conditions with comparable prevalence (Lipinski et al. 2022). Indeed, Nimbley et al. also note a lack of clinician understanding of autism among ED clinicians. When considering that co‐occurring conditions can present additional needs and challenges, it is perhaps not surprising that interventions designed without taking these distinct needs into account may be less helpful for specific populations. As we summarize above and as observed by Nimbley et al., autism as a co‐occurrence with ED is no exception. Autism can present additional challenges and complexities, including differences in communication and social interaction, atypicalities in sensory processing, and a greater need for structure, among other characteristics. These additional needs can contribute to various issues when both seeking and receiving treatment. For instance, social communication challenges may contribute to difficulties in expressing and communicating needs, experiences, and concerns to clinicians. Clinicians may, therefore, misinterpret the concerns of their autistic clients or miss nuanced details, potentially leading to misdiagnosis or less appropriate treatment pathways, particularly where there is a lack of clinician understanding of autism. When receiving interventions, characteristics of autism can also influence the appropriateness of interventions, as well as their understanding and adherence to them, ultimately affecting efficacy. In many cases, interventions and therapeutic approaches have been tailored to address the unique needs of autistic populations. One common example is Cognitive Behavioral Therapy (CBT), often used to address co‐occurring mental health conditions in autism. Here, CBT programs have undergone modifications for use with autistic individuals, aiming to improve their effectiveness and accessibility.

An important point to note when it comes to discussing the development of interventions in the context of autism is the unique context in which autism research and practice are currently situated. The landscape of autism research and practice could be described as in a somewhat tumultuous period, where there are rapidly shifting developments related to the conceptualization of autism and research and intervention approaches. In some contexts, there is a growing divide between the autistic community and clinical care. Autistic individuals are increasingly vocal about the interventions available to them, and there can be conflict between the perspectives and priorities of autistic individuals and those of clinicians. For instance, behavioral approaches such as Applied Behavioral Analysis (ABA), a commonly used intervention approach, have received extensive criticism from some autistic adults (Pukki et al. 2022). Criticism of such interventions may be seen as reflective of larger issues of mistrust, owing to misalignment of goals, a lack of transparency and inclusion of lived‐experience voices, and past interventions that may carry perceived negative effects. Autistic individuals may, therefore, hold reservations about the objectives of clinicians and researchers and be cautious of new interventions where they may perceive that they do not prioritize or align with their needs. Overall, autistic individuals desire input on the interventions that affect them.

In essence, considerations related to intervention in the area are complex but two‐fold. First, findings from Nimbley et al., along with those relating to other co‐occurrences and interventions, suggest that autism‐specific interventions are required. Second, there is a need to bridge the gap between autistic community desires and expectations, and the interventions typically provided within clinical settings. With this context in mind, the question is thus, how do we best move forward to address ED in autistic populations? Nimbley and colleagues begin to allude to such next steps, by proposing a need for more lived experience perspectives. Expanding upon this notion, we propose that the utilization of participatory approaches can help to address the two‐fold issues when it comes to intervention development for autism. First, by ensuring interventions address the unique needs of individuals presenting with co‐occurring autism and ED, and secondly, by ensuring that community and clinical priorities align. Indeed, participatory approaches have been deemed critical by autistic community members (Pukki et al. 2022).

Participatory approaches involve the inclusion of populations of interest within the research process. These approaches can take various forms and be adapted to the needs of specific populations and research questions. Participatory approaches range from consultation with stakeholders, where their input is sought on various components of the research, to true partnership, where researchers and stakeholders work together in equal partnership to co‐create research and its outputs (Vaughn and Jacquez 2020).

Participatory approaches can bring several advantages, foremost, by ensuring that research and its outputs are acceptable, applicable, and beneficial to the populations in which they intend to benefit. Participatory approaches seek to prioritize community input and lived experience expertise, based on the assumption that individuals hold valuable expertise in relation to their own experiences and needs. When applied to ED research and the development of ED interventions, the meaningful engagement of autistic individuals can provide important insights. For example, gathering lived‐experience perspectives can provide insights into the experiences, needs, and nuances associated with co‐occurring conditions that may be otherwise missed by clinicians and researchers. This lived experience input can inform modifications to existing interventions or the development of new interventions. Ultimately, when the experiences of autistic individuals are accounted for during intervention development and considered in combination with treatment efficacy data, the resulting interventions may be more appropriate and acceptable to autistic individuals, in turn improving their effectiveness. Such efforts to gather lived experience input on the experiences of co‐occurring autism and ED and its treatment could also compare findings with other co‐occurring groups where there may be some shared features or treatment approaches.

The meaningful involvement of stakeholders throughout the research process can also help to bridge the divide between community expectations and clinical practice. Involving communities in the research process can help to ensure that the goals of communities, researchers, and clinicians are aligned. It can also contribute to a greater degree of transparency and trust. Again, such benefits ensure that outputs, such as autism‐specific ED interventions, are acceptable and appropriate for the autistic community.

To assist researchers interested in using participatory approaches in ED and ED treatment for autistic populations, we provide a summary of areas in which community members may input across the phases of intervention development (Table 1). Please note, this list is only demonstrative and not exhaustive, and that benefits of participatory approaches can only be seen if community members are genuinely and meaningfully engaged.

**TABLE 1:** Summary of areas in which community members may input across phases of intervention development for ED.

Stage	Participation examples
Conceptualization (pre‐intervention development)	Informing priorities for research and clinical care in relation to ED. Providing insights into unique mechanisms and experiences of ED in autism. These insights could also be compared with those of other co‐occurring groups.
Development (intervention development)	Identifying and defining important intervention targets. Identifying relevant intervention components. Developing and informing the design of intervention approaches and components. Providing feedback on the appropriateness of intervention approaches and their components.
Evaluation	Determining what metrics are important for evaluating ED treatment effectiveness. Informing the selection of research study designs (acceptability to autistic participants). Monitoring, interpreting, and evaluating findings.
Dissemination	Identifying relevant and appropriate dissemination strategies. Dissemination to community groups. Involvement in publications (co‐authorship, intervention manuals).

In conclusion, the review by Nimbley et al. explored the important topic of treatment efficacy for ED in autistic populations, finding evidence to suggest that ED interventions may be less appropriate and effective for autistic populations, and proposing a need for investigation of tailored autism‐specific ED interventions. In this commentary, we discuss these findings in relation to extant literature related to autism, co‐occurring conditions, and intervention development. We suggest that using participatory approaches may present a way forward to improving treatment outcomes for autistic individuals with ED. While we acknowledge that participatory approaches may introduce increased complexities in the research process, acknowledging the experiences of autistic individuals is fundamental to ensuring that resulting interventions are appropriate and acceptable to autistic individuals.

## Author Contributions

**Melissa H. Black:** conceptualization, writing – original draft, writing – review and editing. **Sven Bölte:** conceptualization, writing – review and editing.
